# The UK stand together trial: protocol for a multicentre cluster randomised controlled trial to evaluate the effectiveness and cost-effectiveness of KiVa to reduce bullying in primary schools

**DOI:** 10.1186/s12889-022-12642-x

**Published:** 2022-03-29

**Authors:** Suzy Clarkson, Lucy Bowes, Elinor Coulman, Matthew R. Broome, Rebecca Cannings-John, Joanna M. Charles, Rhiannon Tudor Edwards, Tamsin Ford, Richard P. Hastings, Rachel Hayes, Paul Patterson, Jeremy Segrott, Julia Townson, Richard Watkins, Julia Badger, Judy Hutchings, the Stand Together Team, Mackenzie Fong, Hayley Gains, Helin Gosalia, Anwen Jones, Bryony Longdon, Fiona Lugg-Widger, Siobhan B. Mitchell, Caitlin Murray, Naomi Rose, Holly Whiteley, Katie Taiyari, Melanie Varley, Margiad E. Williams

**Affiliations:** 1 Bangor University, Bangor, Gwynedd LL57 2DG UK; 2 Department of Experimental Psychology, Oxford University, Oxford, OX2 6GG England; 3 Centre for Trials Research, Cardiff University, Neuadd Meirionnydd, Heath Park, Cardiff, CF14 4YS UK; 4 Institute for Mental Health, University of Birmingham, Edgbaston, Birmingham, B15 2TT England; 5 Birmingham Women’s and Children’s NHS Foundation Trust, Steelhouse Lane, Birmingham, B4 6NH England; 6 Department of Psychiatry, Hershel Smith Building, Cambridge Biomedical Campus, Robinson Way, Cambridge, CB2 0SZ England; 7 Centre for Educational Development, Appraisal and Research, University of Warwick, Coventry, CV4 8UW England; 8 Centre for Developmental Psychiatry and Psychology, School of Clinical Sciences at Monash Health, Monash University, Clayton, VIC 3168 Australia; 9 College of Life and Environmental Sciences, Psychology, University of Exeter, Exeter, EX1 2LU England; 10 Birmingham Women’s and Children’s NHS Foundation Trust, Birmingham, UK; 11 Regional School Effectiveness and Improvement Service for North Wales (GwE), Bae Colwyn, UK

**Keywords:** Bullying, Intervention, KiVa, School, Effectiveness, Cost-effectiveness, Economic evaluation, Process evaluation, Cluster randomised controlled trial, School intervention, Violence prevention, Children

## Abstract

**Background:**

Reducing bullying is a public health priority. KiVa, a school-based anti-bullying programme, is effective in reducing bullying in Finland and requires rigorous testing in other countries, including the UK. This trial aims to test the effectiveness and cost-effectiveness of KiVa in reducing child reported bullying in UK schools compared to usual practice. The trial is currently on-going. Recruitment commenced in October 2019, however due to COVID-19 pandemic and resulting school closures was re-started in October 2020.

**Methods:**

Design: Two-arm pragmatic multicentre cluster randomised controlled trial with an embedded process and cost-effectiveness evaluation.

Participants: 116 primary schools from four areas; North Wales, West Midlands, South East and South West England. Outcomes will be assessed at student level (ages 7–11 years; n = approximately 13,000 students).

Intervention: KiVa is a whole school programme with universal actions that places a strong emphasis on changing bystander behaviour alongside indicated actions that provide consistent strategies for dealing with incidents of bullying. KiVa will be implemented over one academic year.

Comparator: Usual practice.

Primary outcome: Student-level bullying-victimisation assessed through self-report using the extensively used and validated Olweus Bully/Victim questionnaire at baseline and 12-month follow-up.

Secondary outcomes: student-level bullying-perpetration; student mental health and emotional well-being; student level of, and roles in, bullying; school related well-being; school attendance and academic attainment; and teachers’ self-efficacy in dealing with bullying, mental well-being, and burnout.

Sample size: 116 schools (58 per arm) with an assumed ICC of 0.02 will provide 90% power to identify a relative reduction of 22% with a 5% significance level.

Randomisation: recruited schools will be randomised on 1:1 basis stratified by Key-Stage 2 size and free school meal status.

Process evaluation: assess implementation fidelity, identify influences on KiVa implementation, and examine intervention mechanisms.

Economic evaluation: Self-reported victimisation, Child Health Utility 9D, Client Service Receipt Inventory, frequency of services used, and intervention costs. The health economic analysis will be conducted from a schools and societal perspective.

**Discussion:**

This two-arm pragmatic multicentre cluster randomised controlled trial will evaluate the KiVa anti-bullying intervention to generate evidence of the effectiveness, cost-effectiveness and scalability of the programme in the UK. Our integrated process evaluation will assess implementation fidelity, identify influences on KiVa implementation across England and Wales and examine intervention mechanisms. The integrated health economic analysis will be conducted from a schools and societal perspective. Our trial will also provide evidence regarding the programme impact on inequalities by testing whether KiVa is effective across the socio-economic gradient.

**Trial registration:**

Trials ISRCTN 12300853 Date assigned 11/02/2020.

## Background

Bullying in childhood is one of the most tractable public mental health problems facing young people [1]. It is generally defined as a pattern of ‘unwanted, aggressive behaviour … that involves a real or perceived imbalance of power’ Olweus [2]. The psychiatric morbidity arising from bullying is substantial; population studies suggest that 25–40% of mental health problems including depression, anxiety and self-harm in young adults may be attributable to childhood bullying [3, 4]. Bullied children access more school health, primary care and specialist child mental health services than their counterparts [5] and experience poor mental health into adulthood [5, 6]. Bullying is also associated with school absenteeism [7, 8] impacting future educational attainment and employment prospects. Children who bully are also at risk of harm and more likely to show later violent behaviour and illicit drug use [9, 10].

This paper describes the protocol for a two-arm pragmatic multicentre cluster randomised controlled Trial (RCT) trial with an embedded process and cost effectiveness evaluation of the Kiusaamisen Vastaan (“KiVa”) anti-bullying programme, the most widely used bullying prevention programme across Europe. In a Finnish RCT (2007–2009) in 234 schools, KiVa significantly reduced bullying and victimisation among 7 to 11-year-old students [11] across all forms of bullying, including verbal, physical, racist, and cyber-bullying [12] and reduced anxiety and depression [13]. Since 2009, KiVa has been scaled up into over 90% of Finnish public schools (approximately 2700 schools) and demonstrated year on year positive effects [14]. A cost-effectiveness analysis reported an increased cost of €829 for a gain of 0.47 victim-free years per student, and a cost per QALY gained of €13,823 considered cost-effective when compared to the Swedish health policy threshold of around €50,000 per QALY [15].

Unlike the UK, Finland’s education system has negligible attainment differences between schools: no selection, tracking or streaming during basic education and highly educated primary school teachers with a mandatory five-year master’s degree qualification [16]. Finland is also one of the wealthiest countries in the world and has less income inequality than the United Kingdom (https://data.oecd.org/inequality/income-inequality.htm). It is, therefore, not certain that KiVa intervention effects will transfer to the UK context. KiVa trials in Italy and the Netherlands [17, 18] found reduced levels of bullying and victimisation with variable effect sizes. A small pilot study in Wales however found no statistically significant reduction in victimization and bullying [19].

The Stand Together RCT is being conducted in UK primary schools with students aged 7 to 11 years, and will include a comprehensive process and health economic evaluation.

## Methods/design

### Aim of study

The Stand Together trial will evaluate the KiVa anti-bullying intervention in the UK to generate evidence of its effectiveness, cost-effectiveness and scalability.

### Trial design

Stand Together is a parallel-group pragmatic, multicentre, two-arm, cluster RCT with a 1:1 allocation ratio of schools and an embedded process and cost-effectiveness evaluation.

Clusters (schools) and students will be recruited across four areas (North Wales, the West Midlands, South East and South West England) with baseline data collected in the 2020/2021 academic year, KiVa implemented in the 2021/2022 academic year, and outcomes measured 12 months post baseline.

### Study setting

The setting is mainstream UK state-maintained primary schools with at least two Key Stage 2 (KS2) classes (4 years of schooling in maintained schools in England and Wales normally known as Years 3–6), for children aged 7–11 years. Primary schools typically serve children aged 4 to 11 years.

### Schools and participants

One hundred and eighteen mainstream primary schools have been recruited (two more than planned).

**Fig. 1:**
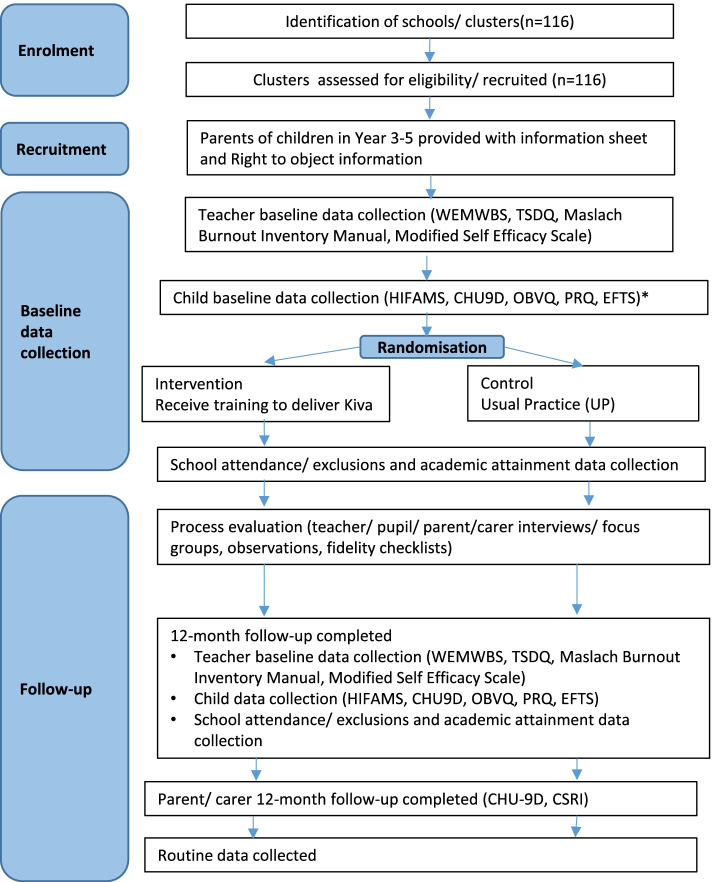
Stand Together Study Flowchart. *Due to school closures in March 2021, a proportion of child baseline data collection was collected after randomisation

Students in Years 3 to 5 provided baseline data from April–July 2021. The cohort of recruited students will provide follow-up data 12-months later when they are in Years 4 to 6 following the delivery of the KiVa programme for one academic year in intervention schools. Data will be collected from all eligible students, and school staff (teachers, higher level teaching assistants, and teaching assistants) in each arm at each time point. See Fig. 1 for the Stand Together study flowchart.

### Inclusion/exclusion criteria

The school inclusion criteria are listed below. There are no inclusion/exclusion criteria for students, with the exception of students whose parents have withdrawn consent, or whose parents did not receive the information sheets with adequate time to consider recruitment documentation.

#### Inclusion criteria are

• Mainstream primary schools with at least two KS2 classes.

#### Exclusion criteria are

- Schools that deliver education through a language other than English or Welsh
- Primary schools already implementing a recognised anti-bullying programme intervention
- Schools that have already implemented KiVa
- Schools that cater solely for students with special educational needs (i.e., Special schools)
- Schools without leadership that can guarantee project participation for the year of data collection/implementation.

### Recruitment and retention

The 118 schools were recruited across the four areas (approximately 30 schools in each area) through professional networks, including educational and anti-bullying agencies that will invite Headteachers/ Governors. Trial participation was offered on a first-come-first-served basis to schools that confirm, in writing, their commitment to the study. Headteachers were informed of all study processes and school requirements during a recruitment meeting with a researcher.

Schools in both the intervention and Usual Practice (UP) conditions will receive a small monetary recompense of up to £308 for time spent by staff in providing research data.

### Informed consent

At recruitment, head teachers were asked to confirm that they had discussed the project with school staff and provide informed consent for their school’s participation in the research. Teaching staff completing self-reported outcomes provided informed consent for their own involvement. Information about the trial was provided to parents and, whilst parents are not able to withdraw their child from the KiVa intervention, they have been given the right to withdraw their child from the research trial and any subsequent data collection. Children will provide their assent for student-level self-report outcomes at each data collection point. If children indicate that they do not assent, either verbally or otherwise through their behaviour, their data will not be collected. Prior to the 12 month follow-up, all parents will receive a letter detailing the plans to link their child’s trial data to data held by the Department for Education, National Pupil Database (NPD). Parents will have the opportunity to opt-out of this activity without it impacting on their child’s continued participation in the trial. For the process evaluation, a sub-set of staff, children and parents will be invited to take part in interviews for which separate consent (for adults) and assent (for children) will be obtained.

### Sample size

The sample calculation was based on previous research (reduction from 18 to 14% in rates of victimisation) [14], and a similar baseline victimisation rate of 18% from a UK based pre-post study [20]. To achieve a 4% absolute reduction (22% relative reduction), 3520 students are required to power an individual level trial at 90% power and a 5% significance level. This equates to an effect size (odds ratio) of 1.35 which is of public health significance and broadly in line with effects achieved from other KiVa trials and the wider literature on bullying and victimisation. The effect size for which this trial is powered is within the mid-range of other relevant KiVa studies, which range from 1.22 [20] to 1.47 [14].

The study will take account of clustering. In the Finnish study [14] the school level intra-cluster correlation (ICC) for the comparison of KiVa and UP in self-reported victimisation was 0.02 at the 12-month follow-up. An average cluster size of 111 students in Years 3 to 5 is assumed, based on recruiting students from three-year groups (years 3, 4, 5) with an average 27.8 students per class (based on 2018 KS2 figures from English schools [21]), and an average 1.34 classes per year group (based on data from the UK KiVa pilot study [20]). A UK study (based on 114 schools) reported an ICC of 0.016 and an average cluster size of 108 Clarkson [20].

Assuming 111 students in Years 3 to 5, an ICC of 0.02, and allowing for one school dropout per arm, 10% student dropout due to either opt-out or loss to follow-up, an 18% rate of victimisation, and a relative reduction of 22%, a trial involving 116 schools (58 per arm) would provide 90% power at a 5% significance level (a total of 12,828 students).

### Randomisation

The 118 schools were randomly allocated by using random permuted blocks on a 1:1 basis to KiVa and UP stratified for KS2 size and percentage of children eligible for free school meals to ensure balance across arms on family socioeconomic disadvantage. Randomisation was carried out by a statistician independent to the study from the Centre for Trials Research, Cardiff University.

### Blinding

Schools were informed of their allocation after teacher baseline data was collected. Whilst all attempts were made to inform schools of their allocation after all children’s baseline data are collected, this was not possible in approximately 60% of schools due to COVID-19 related time constraints. However, there should have been no visible signs or KiVa related changes for the children during the academic year (2020/21). Comparison of data collected prior and subsequent to allocation notification will determine if this had any impact on our primary outcome.

After baseline data collection, students, teachers, trial managers, the intervention delivery team (trainers) and researchers involved in the process and cost-effectiveness evaluation will not be blind to intervention status. It is practically impossible to maintain a blind for fieldworkers as the KiVa interventions includes prominent displays around the schools, but the trial statistician will remain blind to allocation status.

### Intervention

KiVa is an acronym for ‘Kiusaamista Vastaan’ which, translated, means ‘against bullying’; ‘kiva’ is also the Finnish adjective for ‘nice’. KiVa is an evidence-based programme developed in Finland for children aged 7 to 15 years, the age range for children in Finnish comprehensive schools. Its development and evaluation were funded by the Finnish Ministry of Education and Culture in 2006, following the lack of change in bullying prevalence after several legislative changes, over 10 years, requiring schools to have anti-bullying policies. Its development and evaluation were commissioned from Professor Salmivalli and colleagues at Turku University.

KiVa is based on research demonstrating that ‘bystanders’ – children who are present during bullying but not actively involved – can contribute to the maintenance of bullying by assisting or reinforcing the perpetrator’s behaviour, giving them a position of power [22]. Defending the victim, on the contrary, can help to make bullying an unsuccessful strategy for attaining high social status. By influencing the behaviour and norms of all students, the social rewards gained by perpetrators are reduced and, consequently, their motivation to bully.

The KiVa programme is informed by a social architecture model of bullying, which demonstrates the significant roles of the bystanders in supporting or standing against bullying [22]. The KiVa programme has two distinct components; universal and indicated actions. Universal whole-school interventions, promoting school-wide change are most effective at reducing bullying [23] and likely to provide a non-stigmatising approach to prevention [24]. Universal actions at the class and school level to prevent bullying help students to recognise bullying, providing them with safe way of responding to incidents, and teaching them to empathise with, and support, victims. The curriculum is grouped into 3 units for students aged 7–9, 10–12 (KS2) and 13–15 years (KS3) respectively. Other universal actions include posters for school corridors, high-visibility vests for break-time supervisors to highlight the presence of supervision, and a parent’s guide. A trained KiVa team carry out indicated actions using scripted strategies, procedures, and documentation for addressing confirmed cases of bullying with both victims and bullies.

The intervention delivery is over one full academic year, however, the programme is designed to be embedded in ongoing school practice. Although the lesson plans target KS2 classes the programme is introduced to all staff, parents and students and is visible across the school. The manualised curriculum targets students in Years 3 and 4 (Unit 1), and Years 5 and 6 (Unit 2). Each unit contains ten structured 90-min lessons, typically delivered fortnightly as twenty 45-min lessons throughout the school year by class teachers. The curriculum encourages student engagement via oral presentations, role-play, videos, group work and whole class activities. Online games that support lessons can be played at home or in school. A trained KiVa team (2 to 3 staff members) address confirmed bullying incidents using the structured and scripted indicated actions.

Intervention school training will be provided in June 2021 prior to the academic year in which implementation starts. Accredited KiVa trainers at each site will deliver training courses for the intervention schools (approximately 8 schools per training) and offer additional support over the course of the implementation year. Two members of the teaching/management team from each intervention school will attend a local two-day training and then lead school-wide implementation.

### Comparison

Comparison schools will continue with standard anti-bullying practice. The mandatory curricula in Wales and England aim to develop students’ pro-social values and attitudes and empower participation in school and community life as responsible citizens. Comparison schools will continue to use existing methods to cover this curriculum, and to address bullying. No other programme or strategies will be prohibited during the trial, so that there is no interference with UP which may vary across comparison schools (e.g., in terms of curriculum, school policies, skills and interests of teaching staff). This will be recorded via annual checklists describing: a) existing policies and practices to prevent and deal with bullying; and b) any changes to policies and practices during the trial period to enable better understanding of UP and supplement and inform the process and economic evaluations.

### Aims and objectives

The aim is to evaluate the effectiveness of the KiVa anti-bullying programme in UK primary schools, with embedded process and economic evaluations to address the following research questions:

- Is the KiVa programme, over one academic year of implementation, more effective than UP in reducing bullying-victimisation among students aged 7 to 11 years in UK primary schools?
- Is the KiVa programme, over one academic year of implementation, more effective than UP in reducing bullying perpetration and improving students’ mental health, school well-being/connectedness, school attendance and academic attainment, and in improving staff confidence, mental wellbeing, and burn-out in UK primary schools?
- Do the effects of the KiVa programme vary by socio-economic status? Do effects vary by gender?
- To what extent is KiVa implemented with fidelity, and what are the key influences on implementation across school contexts and the relationship between fidelity and outcomes?
- What is the cost-effectiveness of implementing the KiVa programme in primary schools in the United Kingdom?

#### Primary outcome

The primary study outcome is student self-reported victimisation, measured by responses on the Olweus Bully/Victim Questionnaire (OBVQ) [25], the most widely used bullying survey in the world and used in Finland and internationally for KiVa trials, enabling comparison across trials. The OBVQ measures different forms of bullying, including verbal, physical, relational and cyber-bullying. The global item: “How often have you been bullied at school in the last couple of months?” will be used to measure victimisation. Students respond on a five-point scale (0 = “not at all”, 1 = “once or twice”, 2 = “2 or 3 times a month”, 3 = “about once a week”, 4 = “several times a week”). Each item will be analysed continuously and dichotomised with those scoring 2 to 4 classified as victimised and those scoring 0 to 1 as not victimised. This categorisation is conceptual (bullying concerns *repeated* acts), but supported by empirical research showing large and highly significant psychometric differences between these two groups on internalising problems [26].

#### Secondary outcomes

i. Student-self-report outcomes:

1. The OBVQ global item: “How often have you bullied others at school in the last few months?” will measure bullying perpetration. Students respond using the same five-point scale reported above that will be analysed continuously and dichotomised, with scores of 2 to 4 classified as perpetration and scores of 0 to 1 as no perpetration.
2. Subjective student wellbeing in school will be measured using the “How I feel about my school” (HIFAMs) [27]. The survey is comprised of seven items which ask students how they feel about various aspects of school life, for example how they feel when completing their school work or when they are in the playground. This survey will be administered at baseline and at 12-month follow-up.
3. Student empathy will be measured using the Empathy Toward Victim Scale [28]. The seven-item measure asks respondents to rate their level of empathy towards victims on a Likert scale anchored from ‘never’ (0) to always (3).
4. Roles in bullying situations will be measured using the “Participant Role Questionnaire” (PRQ) [28], designed to identify the different roles that peers play in bullying situations. The questionnaire identifies the five different bullying roles; bully, assistant, reinforcer, defender and outsider. The respondent rates how often they behave in the ways described for each role on a three-point scale (Never, Sometimes, Often).

ii. Teacher-report student outcomes: these will be measured through teacher survey reports for each child:

5. Teacher-reported Strengths and Difficulties Questionnaire (TSDQ) [29] will be administered at baseline and follow-up to measure student mental health. These 25-item screening instruments are widely used in developmental, social, clinical and educational studies to detect behavioural, hyperactive/inattentive, emotional, and peer problems and pro-social strengths in children. The standard version asks about behaviours observed over the previous 6 months. The TSDQ is brief, quick to complete, and validated in national UK samples.

iii. Student-level data collected from schools:

6) School administration staff will provide student-level records of authorised and unauthorised half-day school absences for eligible students for the year prior to baseline and during the intervention year. These data are routinely collected by schools for all students as a legal requirement. Schools will ensure that student anonymity is protected.
7) At follow-up, data from the Absence and Exclusion, and KS2 attainment datasets will be requested from the National Student Database (NPD) for students in both trial arms.

iv. Individual staff-level outcomes. We will measure the following secondary outcomes through survey self-reports from teaching staff from KS2:

8) Adapted version of the 5-item Challenging Behaviour Self-Efficacy Scale, designed as a measure of teacher self-efficacy related to challenging behaviours and adapted to specifically refer to bullying behaviours [30].
9) Warwick-Edinburgh Mental Wellbeing Scale [31]. This 14-item positively worded scale measures adult mental wellbeing with good test-retest reliability (*r* = 0.83) and high internal consistency (a = 0.89). This measure will be administered at baseline and at 12-month follow-up.
10) Maslach Burnout Inventory-General Survey (MBI-GS) [32], an introspective 22 item psychological Inventory. It provides a three-dimensional description of emotional exhaustion, depersonalisation, and personal accomplishment.

#### Health economics measures

11) Child reported quality of life (CHU-9D) is a paediatric generic preference-based measure of health-related quality of life with nine dimensions (worried, sad, pain, tired, annoyed, school work/homework, sleep, daily routine and ability to join in activities), each with five response categories, which are scored 1–5 [33, 34]. The self-reported version of the measure, validated for use with children aged between 7 and 17 years old [34], will be administered to students at baseline and at 12-month follow-up.
12) The Client Service Receipt Inventory (CSRI) is a bespoke service use questionnaire that will be used in this study to collect information on the frequency of additional school-based service use by students (e.g., Additional Learning Needs team) and parent-school consultations regarding bullying. Consenting parents will be asked to complete this retrospective CSRI questionnaire at 12-month follow-up. The CSRI will ask for above information for the preceding 6 months and will include associated questions regarding loss of earnings due to bullying-related consultations. CSRIs are widely used in health economics approach to gather information on participant service use. A repository of CSRI questionnaires is available (https://dirum.org/) to assist those developing CSRIs for economic evaluations.

##### Patient and Public Participation (PPI)

This study will use feedback from students, teachers and school leaders to review and improve key aspects of the research, including the trial design and the nature of written communication materials and initial training sessions. The reporting of patient and public involvement in this study will follow the short form Guidance for Reporting Involvement of Patients and the Public (GRIPP2-SF) [35].

#### Process evaluation measures

The MRC guidance on process evaluation will be used as a framework [36]. A mixed methods approach will be adopted. Quantitative methods will assess intervention fidelity, and recruitment of schools, students and parents/carers. Qualitative methods will examine implementation processes, operation of intervention mechanisms, the role of contextual factors and will interrogate patterns in the quantitative data. The quantitative data from the main trial (e.g., on hypothesized mediators) will explore operation of intervention mechanisms.

Implementation fidelity will be defined as the delivery of the main KiVa components (classroom lessons, universal actions at school level, indicated actions to deal with bullying incidents) in line with a minimum level of adherence. KiVa lesson implementation fidelity will be assessed by using data supplied by KiVa coordinators in each school which captures the number of KiVa lessons which each class has delivered. Adherence will be defined as delivery of at least 70% of lessons (of those due to have occurred at the time of reporting) in all classes within a school which are delivering KiVa as part of the trial.

Fidelity of classroom lesson implementation will be explored in further detail in a sample of 16 schools, in which researchers will observe and rate lessons using checklists produced by the KiVa intervention developers. These will assess the extent to which planned KiVa lesson activities are completed, and student learning and engagement. Researcher observation sheets will also include additional questions on the class environment and any challenges encountered during the lesson.

To assess fidelity of universal school level actions, permission will be sought to access existing fidelity checklists completed by KiVa trainers in each intervention school. These cover: a) provision of information on KiVa to staff, students and parents/carers; and b) promotion of the KiVa intervention across the wider school, including use of posters and staff tabards. To assess fidelity of indicated actions, each school will be asked to complete a termly checklist which captures the extent to which bullying incidents have been dealt with in line with the KiVa strategies, procedures and documentation. In each school where observation takes place, two lessons will be observed and where possible researchers will observe two teachers delivering the programme in each of the 16 schools.

**Factors affecting implementation**

In-depth case studies will be conducted in up to eight intervention schools (two per study site) to explore implementation processes, and how these vary across different school contexts. In each school, interviews will be conducted with: the KiVa Coordinator; a member of the KiVa team (which responds to bullying incidents); 1–2 class teachers involved in lesson delivery; and, if applicable other staff involved in intervention delivery. Interviews will explore staff experiences of, and key factors affecting implementation, its perceived value for their professional roles and schools, facilitators of, and barriers to, its integration within school systems and whether adoption of KiVa has led to changes in, or discontinuation of, other activities/policies. Staff will be asked about their receipt of intervention training and support, their confidence in dealing with bullying and the extent to which they perceive their school as having a strategy for bullying following KiVa adoption.

**Intervention mechanisms**

Up to three focus groups will be conducted in each case study school, with students (Years 4–6) who have received KiVa lessons, to examine acceptability, knowledge of KiVa content, and whether it has shaped peer interactions. In case study schools, we will also conduct interviews with approximately five parents/carers to explore the extent to which information about KiVa has been communicated to families, and its receipt by them.

Case study interviews and focus groups will enable us to refine understanding of key intervention mechanisms, and how contextual factors shape their operation. Qualitative findings on mechanisms – and their variation across schools, together with mediation analyses, will help to refine the intervention logic model. Data from case study schools will seek to identify any unintended (positive or iatrogenic) mechanisms.

**Usual practice**

All schools will be asked at recruitment and follow-up to complete a pro-forma (via an interview with a researcher) describing their policies and practices in relation to preventing and dealing with bullying incidents. These data will enable a description of UP that will inform future research. In each study site the key school link member from three control schools will be invited to take part in an interview to further explore UP in relation to bullying.

#### Routinely collected data

At follow-up, participants’ parents will be provided with information and an opportunity to opt their child out from their child’s trial data to be linked with their routine education data. Children’s identifiers: Name, School, Year Group, DoB (where available), Gender (where available) will be provided by sites to enable this linkage. Education data will be requested from the NPD in England and student attainment dataset in Wales via their formal approval processes. Data requested will be related to some demographic information (Ethnicity, special educational needs), attendance, and academic attainment from the Key Stage 2 assessment to enable a comparison between trial arms. Data from the trial will be sent to the ONS Secure Research Service, a secure data safe haven used by the Department for Education and linked on a record level with the data from the NPD using the participant ID.

### Data collection

Table 1 summarises the timetable for data collection. Baseline data was collected in April–July 2021 via student and teacher surveys (teacher-reported measures in March–May and child reported (year 3, 4 and 5 students) measures in April–July 21). The same measures will be collected approximately 12 months post-baseline (May–July 2022) for students in Years 4, 5, and 6. Absence will be collected in September 2021 and September 2022 for prior academic year.

**Table 1:** The Stand Together Participant Timeline (SPIRIT figure): schedule of enrolment, interventions and assessments

Measure	Enrolment	Baseline	Allocation	End of academic year data	Intervention period	Follow-up 12 month post-randomisation
	Dec 20-April 21	April–July 21	May 21	Sept 20/21 Sept 21/22	Sept 21-July 22	May 22–July 22
**ENROLMENT:**						
Headteacher informed consent	X					
Informed of allocation			X			
**INTERVENTIONS:**						
*KiVa intervention-intervention arm only*					X	
*Usual practice-control arm only*					X	
*Usual practice data*	X					X
**PUPIL –REPORTED ASSESSMENTS:**						
How I Feel About My School (HIFAMS)		X				X
Child Health Utility 9D (CHU9D)		X				X
Olweus Bullying and Victimisation Questionnaire (OBVQ)		X				X
Participant Role Questionnaire (PRQ)		X				X
Empathy for the Victim Scale (EFTVS)		X				X
**TEACHER QUESTIONNAIRE:**						
The Strengths and Difficulties Questionnaire (TSDQ)		X				X
Maslach Burnout Inventory Manual		X				X
Modified Self Efficacy Scale		X				X
The Warwick – Edinburgh Mental Well-being Scale (WEMWBS)		X				X
School attendance, exclusions and academic attainment				X		
Process evaluation-teacher interviews/ focus groups					X	
Process evaluation-teacher interviews/ focus groups					X	
Routine data						X
Child Health Utility-9D (CHU-9D)						X
Client Service Receipt Inventory (CSRI)						X

### Data management and security

Student and teacher names were collected and stored securely on password protected folders at respective research sites, separate from any trial data. Student-reported and teacher-reported trial data are being entered on to an offline Qualtrics application system on tablet screens in the first instance, with a paper version of each questionnaire (to be subsequently entered onto the Qualtrics database) as a back-up in case of any technical issues or school request. Pseudonymised trial data will be uploaded to Qualtrics and exported to Excel or SPSS for data checks and cleaning prior to data analysis. All paper CRFs will be stored at sites, prior to being transferred by courier to the CTR. Paper CRFs will be stored securely in locked cupboards and all electronic data will be stored securely on password-protected secure Cardiff University servers. Data transfer to site researchers for additional analysis will be via the Cardiff University secure Fastfile system.

Qualitative interview and observation recordings will be recorded on encrypted audio-recorders/video recorders and stored on password-protected computers at sites. Recordings will be securely transferred to the study team at the CTR by Fastfile or courier. All files will be encrypted, and transcripts will be fully pseudonymised prior to analysis. All qualitative interviews will be audio-recorded, transcribed fully, and pseudonymised for analysis. Computer software (NVivo) will be used to manage the qualitative data and transcripts.

Data security and confidentiality will be ensured, in line with GDPR. A data management plan will be completed and adhered to. Only the trial team will have access to the final study dataset.

### Data monitoring

An initial internal pilot phase assessed the feasibility of recruitment. An independent Trial Steering Committee (TSC) is overseeing the trial. The TSC determined at their first meeting that no separate Data Monitoring and Ethics Committee (DMEC) was required, as the TSC would also fulfil this remit.

Progression criteria were as follows:

Trial feasibility was based on recruitment of schools and students across the four sites with progression to a full trial in all sites dependent upon progress. Progression thresholds are set out in Table 2: 1) Green: continue to full trial without modification; 2) Amber: remediable issues; proceed with caution following review processes with the Trial Steering Committee and PPI group to; Red: early cessation, intractable issues. The decision to continue was made in May 2021.

**Table 2:** Progression criteria for the Stand Together Trial

	Green	Amber	Red
% of target schools recruited	≥90%	80–89%	≤79%
% eligible students recruited	≥80%	60–79%	≤59%
% baseline primary outcome collected	≥90%	80–89%	≤79%
% of baseline secondary outcomes collected	≥80%	65–79%	≤64%

### Statistical methods

A detailed statistical analysis plan will be written prior to database lock. We will report trial outcomes following CONSORT guidelines for cluster RCTs [37] and the SPIRIT statement for trials of interventions [38]. This will involve a combination of quantitative and qualitative methods. Statistical analysis will be performed in Stata (version 63 or higher). All analyses will be intention to treat (ITT) (i.e., students will be analysed in the groups to which they were randomised, regardless of adherence to intervention) and missing outcome data will not be replaced (all main statistical analysis will be conducted on complete cases). Between-group comparisons will be presented with two-sided 95% confidence intervals (CI). As the trial includes a reasonable number of clusters, the analysis will be based on the individual student, allowing for clustering between students within school using robust standard errors. Analyses will control for the school level stratification variables (geographical area, school size, proportion of students eligible for Free School Meals (FSM)). Key student characteristics (age, sex) will also be controlled for.

#### Primary outcome

Multilevel logistic regression models will be used to compare the proportion of students reporting victimisation at 12 months post randomisation by arm, and results presented as odds ratios and 95% CIs. ICCs alongside 95% CIs will also be reported.

#### Secondary outcomes

Multilevel linear (continuous outcomes) and logistic (binary outcomes) regression models will be used to compare secondary outcomes.

#### Sub-group analyses

These will investigate the effect of the intervention by student sex and FSM status. Interactions will be modelled between the study arm and these variables. The results of these exploratory analyses will be presented using 95% CIs.

#### Missing data

To investigate the impact of any missing outcome data on the trial conclusions, missing mechanisms will be explored, and appropriate imputation methods applied via sensitivity analyses. Baseline characteristics will be compared with post-randomisation variables of students who have and have not completed primary outcome data. Multiple imputation will be performed to assess the impact of missing outcome data using the mi command in Stata. Imputation models will include outcomes (including student attendance), intervention arm, stratifying variables, and a main school effect to allow for clustering, as well as any appropriate baseline covariates. The main analyses will be repeated on the imputed datasets. As an added sensitivity analysis, a scenario-based imputation will be carried out such as best-worst case scenario assuming all lost to follow up in the KiVa arm have been victimised and vice versa.

#### Intervention compliance

The effect of KiVa lesson dosage on the primary outcome will be explored in a sensitivity analysis and estimated in a way that preserves randomisation using Complier-Average Causal Effect (CACE) estimates, using a two-stage least squares instrumental variable regression model Dunn [39].

#### Qualitative analysis

With appropriate consent, all interviews and focus groups will be audio-recorded, transcribed fully, and pseudonymised for analysis. Computer software (NVivo) will be used to manage the qualitative data and transcripts. Thematic analysis [40] will be used to analyse each of the sub-sets of interviews/focus groups (school staff, pupils, parents/carers) separately and independently. A thematic analysis approach will be used for a qualitative synthesis across the interview/focus group sub-groups. This analysis will then provide an over-arching synthesis of school staff, pupils’ and parents/carers’ experiences and perceptions related to the process evaluation aims. Where appropriate (e.g., where both school staff and parents/carer discuss the same issue – e.g., school context) we will examine the data and the extent to which the views of these groups are aligned.

A Qualitative Analysis Plan (QAP) will provide further detail on the approach and steps which will be taken to the analysis of the qualitative data from the process evaluation.

#### Triangulation of qualitative and quantitative data

A triangulation exercise will be conducted combining all of the qualitative results with the quantitative data analysis results including school recruitment, implementation fidelity, intervention mechanisms and their interaction with local context. Data collection across the study will be designed to maximise the potential for triangulation.

We will draw on Palinkas et al’s (2011) taxonomy of mixed methods designs to guide triangulation of data collection and analysis [41].

### Cost-effectiveness

The full cost of delivery of the KiVa intervention and associated costs will be calculated. The health economic analysis will be conducted from a schools and societal perspective [42–44].

Taking into account National Institute of Health and Care Excellence guidance on economic evaluations of public health interventions [45] and the effects of clustering [46], we will undertake a primary cost effectiveness analysis of the KiVa intervention, using student self-reported quality of life (CHU-9D) as the outcome of effect [42–44]. Costs calculations will include the time and resources required to implement KiVa in schools, including the cost of any training. This information will be collected from CEIT, the UK KiVa programme trainers, and KiVa team members in intervention schools. Staff salary costs will be calculated using national sources (National Union of Teachers Salary Card, 2018) [47]. Cost and quality of life data will subsequently be combined to calculate an incremental cost-effectiveness ratio (ICER), which will report the cost per unit of effect (i.e., cost per quality adjusted life year using KiVa compared to the control condition) [42–44]. We will also be interested in the proportion of children moving from “bullied” to “not bullied”, over the study period in each condition compared with the marginal cost of delivery of KIVA in schools.

We will also embed a wider cost consequence analysis [42] to explore self-reported bullying incidences using OBVQ data along with other outcome measures collected for both students and teachers [25].

A fully documented Health Economics Analysis Plan (HEAP) will be written based on published guidance for best practice [48], aligning with the Statistical Analysis Plan, and agreed by the co-applicants before follow-up data collection has been completed, as the HEAP is intended as a living document to incorporate any changes or updates as the trial progresses. This plan will be locked before any analysis is undertaken. The cost-effectiveness analysis will be reported according to the Consolidated Health Economics Evaluation Reporting Standards.

### Ethical approval and consent to participate

Ethical approval was granted by Bangor University Psychology Research Ethics and Governance Committee (2019–16,592) on 13th November 2019.

### Amendments to ethical approval

There have been 12 minor amendments to our original Ethics application, which include minor revisions/additions to information sheets and consent forms, minor changes to the protocol such as the addition of questionnaires for teachers and the inclusion of covid procedures as well as forms for the process evaluation element of the trial. There was also the inclusion of a sub-study to explore pupils’ social and emotional wellbeing and changes in bullying behaviour and relationships over time. The list of amendments with approval dates are as follows:

**Table:** 

2019–16,592	Original application approved 13/11/19
2019–16,592-A14604	Amendment approved 28/11/19
2019–16,592-A14613	Amendment approved 17/12/19
2019–16,592-A14627	Amendment approved 27/01/20
2019–16,592-A14631	Amendment approved 27/01/20
2019–16,592-A14662	Amendment approved 24/03/20
2019–16,592-A14668	Amendment approved 09/04/20
2019–16,592-A14716	Amendment approved 16/11/20
2019–16,592-A14730	Amendment approved 04/01/21
2019–16,592-A14761	Amendment approved 23/03/21
2019–16,592-A14786	Amendment approved 07/07/21
2019–16,592-A14802	Amendment approved 13/09/21
2019–16,592-A14843	Amendment approved 25/11/21

### Benefits and risks

If successful, the Stand Together trial will result in the following benefits:

- Reduction of bullying, which will be of benefit to all participants, victims and bullies, the whole school, local community, and society in general.
- Reductions in poor mental health (enhanced emotional well-being) and improved quality of life for students.
- Evidence on the costs and cost effectiveness of the KiVa programme in the UK.
- Evidence regarding programme impact on inequalities by testing whether the intervention is effective across the socio-economic gradient.
- Benefits to school staff through increased training and improved school environment, which may improve staff well-being.
- Benefits to students who participate in the intervention through opportunities for learning and improved self-efficacy.

There are no anticipated risks to participants or schools. However, there may be unanticipated events and we will actively seek evidence of any adverse effects in the process evaluation and trial data. Should any member of the research team become concerned at any point about issues in relation to any individual student, staff will follow a study-specific Standard Operating Procedure for dealing with harm that will be explained to head-teachers during the consent process and highlighted explicitly in school information sheets.

The introduction of KiVa may replace the use of other Personal Social Education (PSE) strategies or interventions to reduce bullying. However, KiVa has been trialled in several countries (e.g., Finland [11], Italy [17] and the Netherlands [18] and found to be effective (with varying levels of effectiveness), and it is unlikely to be detrimental. KiVa is presently one of only four bullying interventions with a Blueprints for Violence Prevention classification as having evidence of effectiveness (http://blueprintsprograms.com). The risks are therefore considered minimal and the anticipated benefits justify the risks.

### Study management and governance

#### Trial documentation

Relevant trial documentation will be kept for a minimum of 15 years.

#### Trial registration and conduct

The trial is registered with http://www.controlled-trials.com (ISRCTN 12300853). We will follow the UK Medical Research Council Guidelines on Good Clinical Practice in Clinical Trials.

#### Sponsor

Bangor University, the employer of one of the co-Principal Investigators Judy Hutchings (JH), will act as the Sponsor of this trial.

Trial Steering Committee (TSC): the trial is being overseen by a TSC, including an independent chair with expertise in school-based research and bullying, and other independent members including a researcher with expertise in bullying, a Health Economist, a Statistician, National Antibullying Organisation, a parent, and Headteacher and non-independent members. The TSC will meet annually throughout the trial and additional meetings will be organised when and if required.

#### Data monitoring

The TSC determined at their first meeting that a separate Data Monitoring and Ethics Committee (DMEC) is not required, and that the TSC will also fulfil this remit.

#### Study management

JH will direct the study together with Lucy Bowes (LB) as co-CI. The intervention and research teams will be functionally independent. The research team will be managed by JH, LB, Richard Hastings (RPH), Tamsin Ford (TF), Rachel Hayes (RH), and Suzy Clarkson (SC). Rhiannon Tudor Edwards (RTE) and Rhiannon Tudor Edwards (RTE) will direct the cost-effectiveness evaluation. Jeremy Segrott (JS) will direct the process evaluation. Fiona Lugg-Widger (FLW) will lead the routine data component of the trial. The trial will be fully coordinated by the Centre for Trials Research (CTR), managed by Julia Townson (JT) and Elinor Coulman (EC). Rebecca Cannings-John (RCJ) will oversee the statistical analysis. Richard Watkins (RW) and SC will oversee the PPI. The trial manager will have day-to-day responsibility for the conduct of the trial and the operations of the research team and will report monthly to the trial management group. The trial management group will meet monthly throughout the study. Responsibility for data integrity and analysis will be held by the Clinical Trials Unit (CTU) at Cardiff University.

#### Audits and inspections

The trial is participant to inspection by the Health Technology Assessment (HTA) programme as the funding organisation. The trial may also be participant to inspection and audit by Bangor University under their remit as sponsor.

#### Dissemination

All publications and presentations relating to the trial will be detailed in the publication policy which has been drafted and authorised by the TMG.

### Trial status

The trial is currently on-going. Recruitment commenced in October 2019, however due to COVID-19 pandemic and resulting school closures was re-started in October 2020. The protocol has been written according to the Standard Protocol Items: Recommendations for Interventional Trials (SPIRIT) statement, and the final report will follow the Consolidated Standards of Reporting Trials (CONSORT) statement.

## Data Availability

Not applicable.

## Abbreviations

CHU-9D: Child Health Utility-9D
CI: Confidence Interval
CSRI: Client Service Receipt Inventory
DMEC: Data Monitoring and Ethics Committee
EIWT: Early Intervention Wales Training
FSM: Free School Meal
HEAP: Health Economics Analysis Plan
HIFAMS: How I feel about school questionnaire
ICC: Intra-Cluster Correlation
MBI-GS: Maslach Burnout Inventory - General Survey
NPD: National Student Database
OBVQ: Olweus Bully/Victim Questionnaire
PRQ: Participant Role Questionnaire
PSE: Personal Social Education
PSHE: Personal Social Health Education
RCT: Randomised Controlled Trial
TLR: Teacher Lesson Record
TSC: Trial Steering Committee
TSDQ: Teacher Strengths and Difficulties Questionnaire
UP: Usual Practice
